# Neuroendoscopy Versus External Ventricular Drainage for Thalamic Hemorrhagic Stroke: A Systematic Review and Bayesian Meta‐Analysis

**DOI:** 10.1002/brb3.71526

**Published:** 2026-06-21

**Authors:** Pedro Tchicama Sikembi, Pablo Andrés Vega‐Medina, Albe Dias Batista, Davi Ricardo Soares Gama de Amorim, Angel F. Godina‐Sanchez, Laura Alexandra González‐Chang

**Affiliations:** ^1^ Faculdade De Medicina Universidade José Eduardo Dos Santos Huambo Angola; ^2^ Ciudad de La Salud, Caja de Seguro Social Panama City Panamá; ^3^ Neuroanatomy Department, Faculty of Medicine, School of Medicine University of Panamá Panama City Panama; ^4^ Escola de Ciências de Saúde Universidade de Amazonas Manaus Amazonas Brazil; ^5^ Faculty of Medicine University of Pernambuco Recife Pernambuco Brazil; ^6^ Faculty of Medicine Universidad de Guadalajara Guadalajara Jalisco Mexico; ^7^ Faculty of Medicine University of Panama Panama City Panama

**Keywords:** Bayesian meta‐analysis, external ventricular drainage, neuroendoscopy, thalamic hemorrhage

## Abstract

**Background:**

Neuroendoscopy and external ventricular drainage are commonly considered first‐line options for managing thalamic hemorrhage. However, their effectiveness in reducing the risk of rebleeding remains unclear.

**Aim:**

To compare the efficacy and safety of neuroendoscopy versus external ventricular drainage through a systematic review and Bayesian meta‐analysis of randomized and nonrandomized studies.

**Methods:**

We searched PubMed, Embase, and Cochrane for randomized and nonrandomized studies comparing neuroendoscopy with external ventricular drainage in patients with spontaneous thalamic hemorrhage. Data analysis was conducted using a Bayesian random‐effects model, with 95% credible intervals (CrI), estimating risk ratios for binary endpoints and mean differences for continuous endpoints.

**Results:**

Six studies comprising 399 patients were included in the meta‐analysis. Although the pooled analysis showed no significant difference between groups in rebleeding risk (RR 0.61, 95% CrI 0.26–1.46; *I* ^2^ = 20.3%), neuroendoscopy was associated with an 87.4% posterior probability of reducing rebleeding risk compared with external ventricular drainage. Notably, neuroendoscopy significantly reduced the need for ventriculoperitoneal shunt (RR 0.48, 95% CrI 0.25–0.91; *I* ^2^ = 23.4%), with a 98.6% posterior probability of benefit. It was also associated with favorable prognosis (RR 1.42, 95% CrI 1.05–1.94, *I* ^2^ = 17%), with a 98.84% posterior probability of benefit.

**Conclusion:**

In patients with thalamic hemorrhage, neuroendoscopy was associated with a higher probability of reducing both rebleeding risk and the need for ventriculoperitoneal shunt compared with external ventricular drainage.

## Graphical abstract

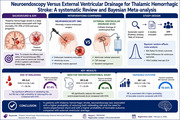

## 1 Introduction

Thalamic hemorrhage remains one of the most challenging forms of intracranial hemorrhage, given the significant difficulties in performing blood evacuation due to the deep location of the thalamus at the base of the cranium (Sreekrishnan et al. 2016). It accounts for 10%–15% of intracranial hemorrhages and is associated with high mortality (Qureshi et al. 2009).

Neuroendoscopy and external ventricular drainage (EVD) are commonly considered first‐line options for managing thalamic hemorrhage (Nam et al. 2021). Despite increasing evidence supporting the effectiveness of neuroendoscopy, the risk of rebleeding remains controversial in both randomized and observational studies (Chen et al. 2021; Yang et al. 2024). EVD is routinely performed when thalamic hemorrhage extends into the ventricles; however, poor prognosis has been reported in cases of severe intraventricular hemorrhage (Gaberel et al. 2012; Nam et al. 2021). Estimating the probability that these interventions reduce unfavorable outcomes is essential for clinical decision‐making.

Although recent frequentist meta‐analyses have reported favorable prognosis and lower mortality with neuroendoscopy compared with EVD in this population, rebleeding risk remained comparable (Pramatya et al. 2025). Additionally, the posterior distribution of heterogeneity and its relationship to effect size have not yet been studied. Therefore, we aimed to compare the efficacy and safety of neuroendoscopy and EVD through a systematic review and Bayesian meta‐analysis of randomized and nonrandomized studies.

## 2 Methods

### 2.1 Study Protocol and Registration

This systematic review and meta‐analysis was conducted in accordance with the Preferred Reporting Items for Systematic Reviews and Meta‐Analyses (PRISMA) guidelines (Page et al. 2021), the Cochrane Handbook for Systematic Reviews of Interventions (Higgins 2024), and recommendations for Bayesian meta‐analysis (Kruschke 2021). The protocol was registered in PROSPERO (CRD420261283309) prior to initiating the screening phase and is available at: https://www.crd.york.ac.uk/PROSPERO/view/CRD420261283309.

### 2.2 Eligibility Criteria

Studies were included if they met the following criteria: (1) randomized or nonrandomized design; (2) comparison of neuroendoscopy with external ventricular drainage (EVD); (3) patients with thalamic hemorrhagic stroke with or without ventricular involvement; and (4) reporting at least one outcome of interest. We excluded (1) studies reporting only outcomes in the overall population of intracranial hemorrhagic stroke, (2) inaccessible studies, and (3) case series with fewer than five patients.

### 2.3 Sources and Search Strategy

A comprehensive literature search was conducted in PubMed, Embase, and Web of Sciences on January 3, 2026. The search strategy combined the following terms: (“Intracranial Hemorrhages” [Mesh] OR “Thalamic Hemorrhage” OR “Thalamic Hematoma” OR “Thalamic Stroke” OR “Thalamic Bleed”) AND (“Minimally Invasive Surgery” OR “Minimally Invasive Surgical Procedures” [Mesh] OR “Neuroendoscopy” [Mesh] OR “Neuroendoscopic Surgery” OR “Smartphone‐Assisted Endoscopic Surgery”) AND (“Ventricular Drainage” OR “EVD” OR “Ventriculostomy” [Mesh] OR “External Ventricular Drainage”). Further details are available at Table S1.

### 2.4 Study Selection and Data Extraction

Study selection was independently performed by two reviewers (AG and LG) using Rayyan (2024 version) to deduplicate records, screen titles and abstracts, and assess full texts for eligibility. Disagreements were resolved by consensus with a third author (ADB).

Data extraction was conducted by two independent authors (DBA and PAVM) using a shared online spreadsheet. Extracted data included study characteristics and outcomes such as study design, mean Glasgow Coma Scale scores, follow‐up time, sample size, number of events, mean age, surgical techniques, and intracranial hemorrhage volume.

### 2.5 Quality Assessment

Risk of bias was independently assessed by two authors using the Cochrane tools for randomized studies (RoB 2) and nonrandomized studies (ROBINS‐I). Disagreements were resolved by consensus. Publication bias was evaluated using funnel plots and confirmed with Egger's test when ten or more studies were available. A summary of findings table was prepared according to the *Grading of Recommendations Assessment, Development and Evaluation (GRADE)* framework.

### 2.6 Study Endpoints

The primary outcomes of interest were:

- Rebleeding, defined as any clinically or radiologically confirmed thalamic hemorrhage during follow‐up.
- Favorable prognosis, defined as a modified Rankin Scale (mRS) score ≤3.
- Mortality, defined as any death during the follow‐up period.

### 2.7 Data Analysis

Data analysis was conducted using a Bayesian random‐effects model, with 95% credible intervals (CrIs) and risk ratios for binary endpoints. Study heterogeneity was quantified using the *I* ^2^ statistic and reported as posterior estimates of the heterogeneity parameter (*τ*) with corresponding 95% CrIs. Prediction intervals for the pooled effect size were calculated to contextualize findings for future studies. Posterior distributions were estimated with 10,000 direct sampling. A Bayesian approach was selected due to the small sample sizes in primary studies and its robustness in estimating between‐study heterogeneity and its ability to provide probability estimates under this framework. Additionally, previous frequentist meta‐analyses have failed to generate significant effect in rebleeding risk, rising concerns in the effectiveness of neuroendoscopy in thalamic hemorrhagic stroke. A Bayesian approach may provide a differential information thereby estimating the posterior probability of benefit associated with neuroendoscopy.

The primary analysis employed vague priors, assuming the log risk ratio followed a normal distribution with a mean of 0 and a standard deviation of 2. Sensitivity analyses were conducted using weakly informative priors to ensure results were not dependent on prior specification. An additional sensitivity analysis using a frequentist random‐effects model was performed for comparison. When sufficient randomized trials were available, a sensitivity analysis restricted to randomized evidence was conducted.

The posterior distribution of the treatment effect was characterized using its probability density function (PDF), providing a continuous representation of the uncertainty around the estimated effect size. The cumulative distribution function (CDF) was used to compute posterior probabilities of clinically relevant thresholds, including the probability that the risk ratio (RR) was less than 1 (or the standardized mean difference [SMD] was less than 0), indicating a beneficial effect. The joint posterior distribution of heterogeneity and effect size was estimated to assess their relationship and identify areas of high precision. Both effect size and heterogeneity distributions were visualized using histograms. All statistical analyses were performed in R using the *Bayesmeta* package

## 3 Results

### 3.1 Study Selection and Baseline Characteristics

**FIGURE 1:**
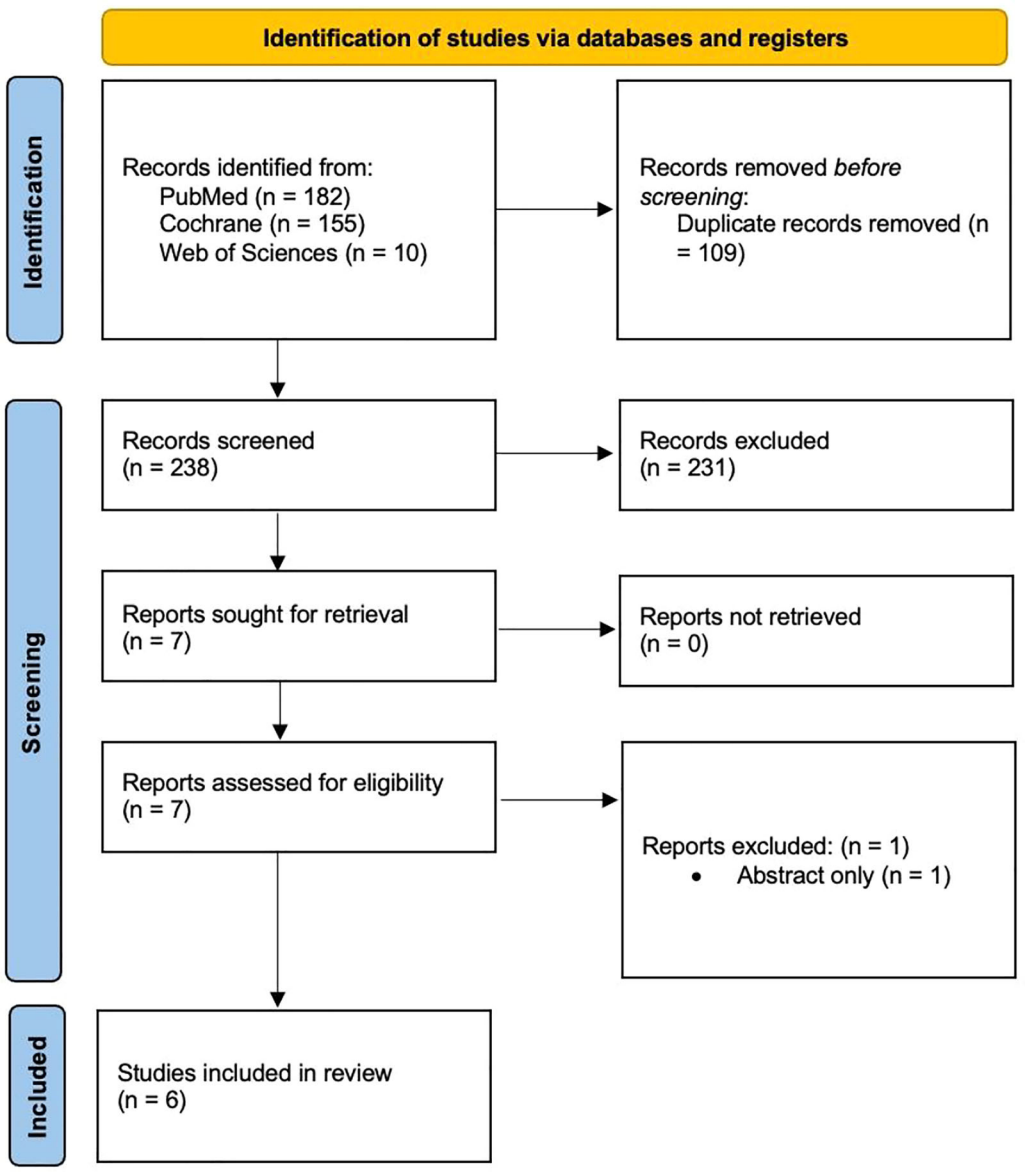
PRISMA flow diagram illustrating study identification, screening, eligibility assessment, and inclusion in the quantitative synthesis.

Database searches yielded 347 records, of which 109 duplicates were removed prior to screening. After title and abstract screening, 231 records were excluded, leaving seven full‐text articles for eligibility assessment. Six studies comprising 399 patients were included in the meta‐analysis (Chen et al. 2011; Chen et al. 2021; Fu et al. 2019; Ge et al. 2019; Yang et al. 2024; Zhou et al. 2022) (Figure 1).

The neuroendoscopy group included 195 patients with a mean age of 61.9 years, while the external ventricular drainage (EVD) group comprised 204 patients with a mean age of 62.9 years. In studies reporting sex distribution (*n* = 311), 162 patients (52.1%) were male. Reported admission Glasgow Coma Scale (GCS) scores ranged from 5.8 to 9.8. Mean hematoma volumes ranged from 10 to 35 mL. Follow‐up ranged from 3 to 12 months (Table 1).

**TABLE 1:** Baseline characteristics of included studies.

Study	Country	Type	Group	*n*	Mean age	Male *n* (%)	Vol. (mL)	Mean GCS	Follow‐up
Yang et al. (2024a,b)	China	Retro	Neuroendoscopy	35	66.37 ± 6.62	19 (54.3)	31.0 ± 2.05	8 (8–9)	6 months
			EVD	40	68.75 ± 7.22	22 (55.0)	30.2 ± 2.30	8 (8–9)	
Zhou et al. (2022)	China	RCT	Neuroendoscopy	41	61.3 ± 3.9	20 (48.8)	NR	5.85 ± 0.21	6 months
			EVD	42	62.1 ± 4.3	22 (52.4)	NR	5.65 ± 0.19	
Chen et al. (2021)	Taiwan	Retro	Neuroendoscopy	7	66.6 ± 10.5	4 (57.1)	35 ± 8.5	NA	12 months
			EVD	7	68.2 ± 12.4	2 (28.6)	18 ± 8.5	NA	
Fu et al. (2019)	China	Retro	Neuroendoscopy	68	58.5 ± 2.12	35 (51.5)	10–30	7–8	6 months
			EVD	71	60.5 ± 0.71	38 (53.5)	10–30	8	
Ge et al. (2019)	China	Non‐RCT	Neuroendoscopy	20	61.0 ± 8.5	NR	35.2 ± 7.4	7.4 ± 2.3	6 months
			EVD	20	59.9 ± 8.7	NR	34.8 ± 8.4	7.7 ± 1.7	
Chen et al. (2011)	Taiwan	RCT	Neuroendoscopy	24	65.54 ± 11.70	NR	10.5 ± 10.7	8.54 ± 2.8	3 months
			EVD	24	62.17 ± 10.74	NR	11.5 ± 9.6	9.83 ± 3.1	

EVD, external ventricular drainage; NR, not reported.

### 3.2 Pooled Results

#### 3.2.1 Rebleeding

**FIGURE 2:**
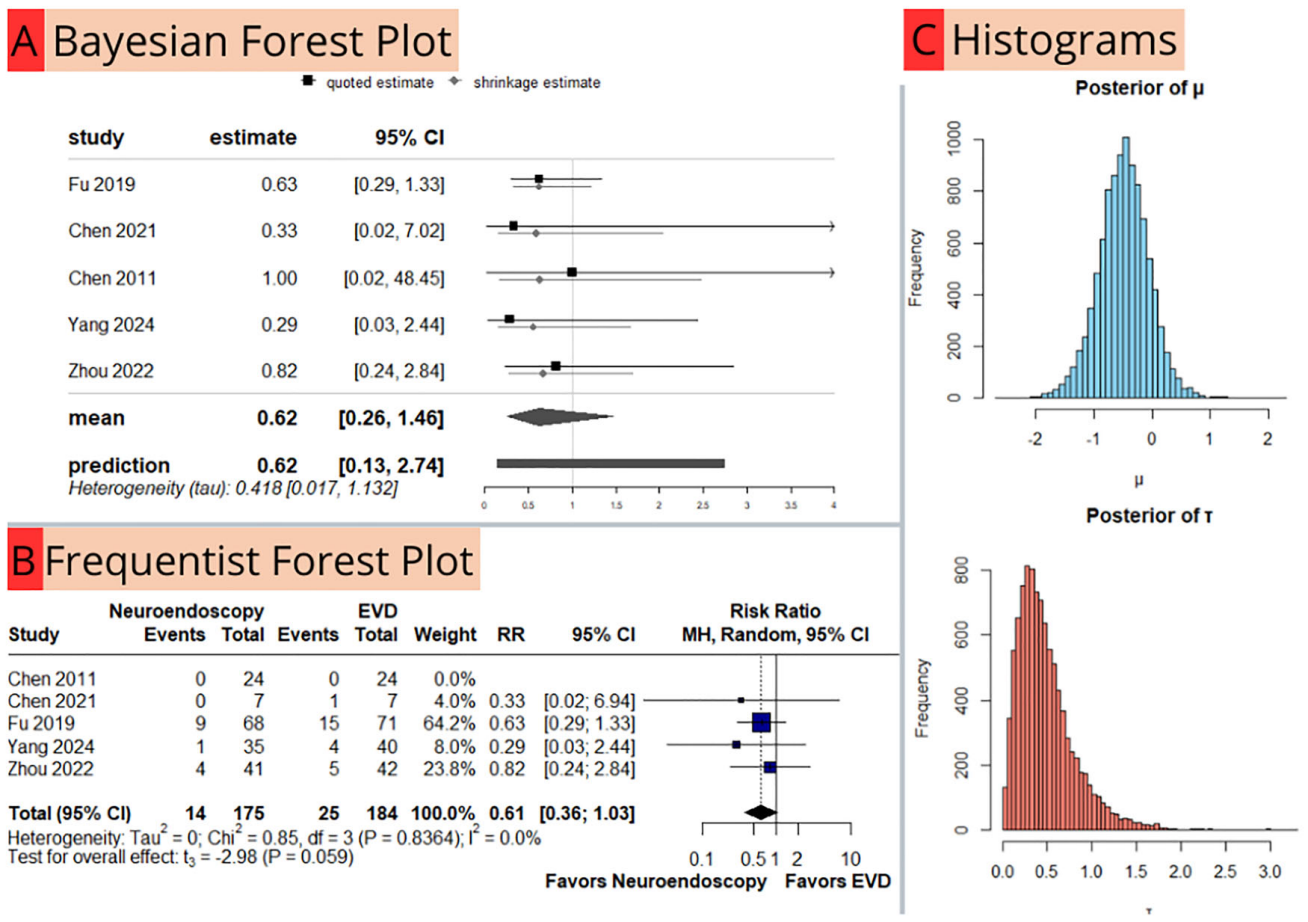
Rebleeding outcomes comparing neuroendoscopy versus external ventricular drainage: Bayesian and frequentist meta‐analysis. (A) Bayesian forest plot showing study‐level and pooled risk ratios (RR) with 95% credible intervals. (B) Frequentist random‐effects forest plot presenting study‐specific and pooled RR with 95% confidence intervals. (C) Posterior distributions from the Bayesian model for the overall treatment effect (*μ*) and between‐study heterogeneity (*τ*).

**FIGURE 3:**
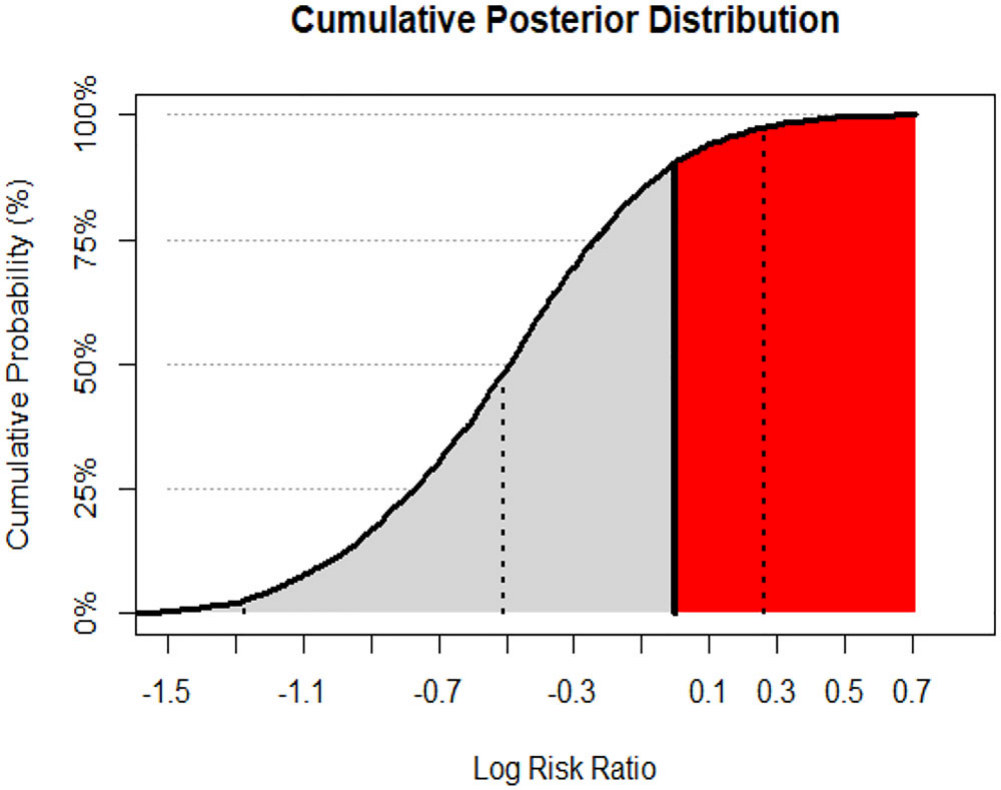
Cumulative posterior distribution of the pooled log risk ratio for rebleeding. The shaded region represents posterior probability mass favoring neuroendoscopic surgery relative to control.

The pooled analysis of five studies showed no clear difference in rebleeding risk between groups, although results tended to favor neuroendoscopy (posterior RR 0.61, 95% CrI 0.26–1.46) (Figure 2). Heterogeneity was low (posterior median *I* ^2^ = 20.3%) (Figure S2). neuroendoscopy demonstrated an 87.4% posterior probability of reducing rebleeding risk compared with EVD, with a symmetric posterior distribution of the estimated log risk ratio centered on the median (Figures 3 and S1).

#### 3.2.2 Mortality

The pooled estimate suggested a lower mortality risk with neuroendoscopy compared with the EVD group (posterior RR 0.61, 95% CrI 0.30–1.27) (Figure S5). Heterogeneity was low (posterior median *I* ^2^ = 24.7%) (Figures S5 and S10), and the posterior probability of benefit for neuroendoscopy was 90.9% (Figures S8 and S9). The frequentist analysis yielded similar results (RR 0.59, 95% CI 0.30–1.16; *I* ^2^ = 0%) (Figure S6).

#### 3.2.3 Prognosis

**FIGURE 4:**
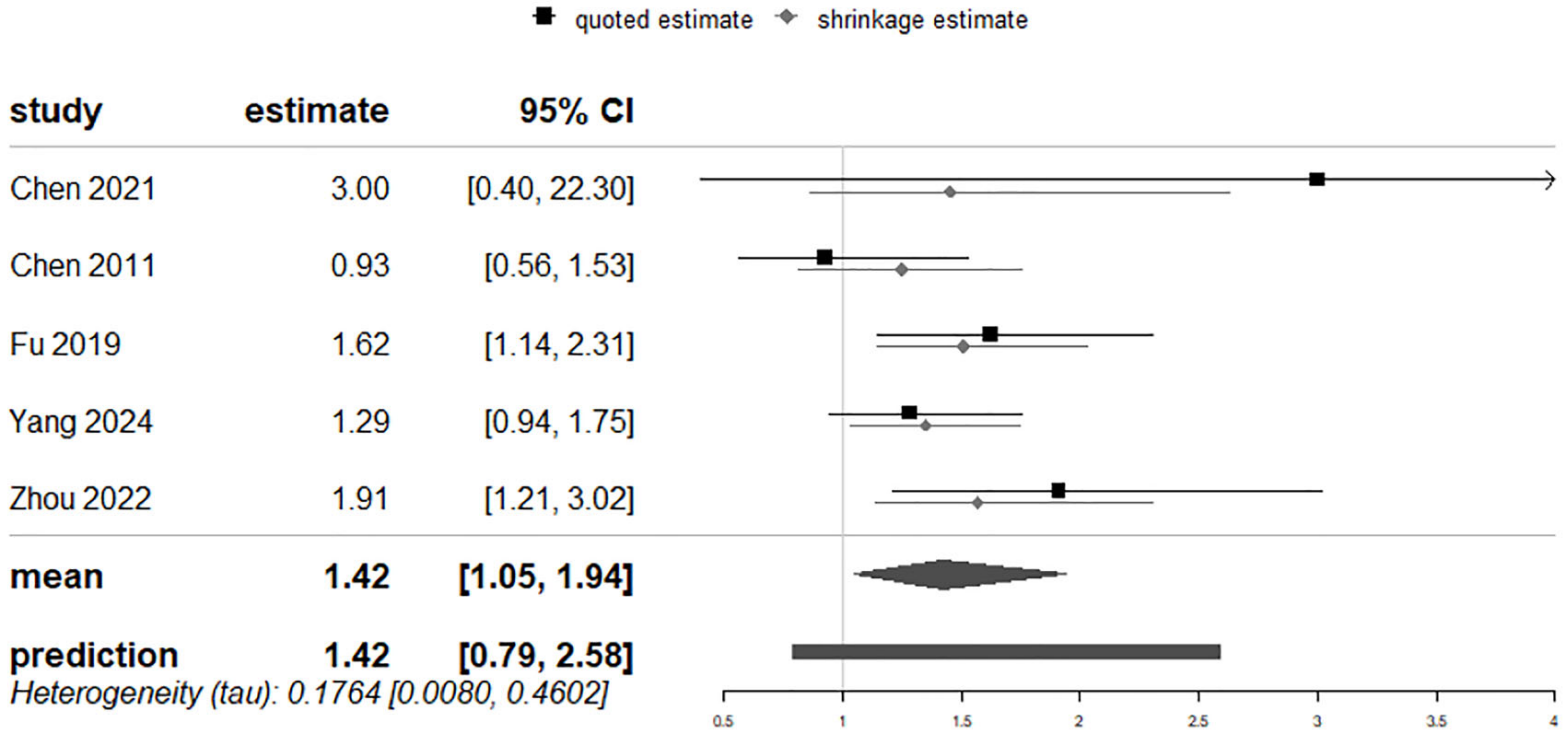
Bayesian forest plot comparing neuroendoscopy versus external ventricular drainage for rebleeding.

Analysis of five studies demonstrated that neuroendoscopy was associated with improved prognosis compared with external ventricular drainage (EVD) (posterior RR 1.42, 95% CrI 1.05–1.94) (Figure 4). Heterogeneity was low (posterior median *I* ^2^ = 17%) (Figures 4 and S14). The posterior probability of benefit for neuroendoscopy was 98.84%, indicating a definitive advantage of neuroendoscopy over EVD (Figures S12 and S13).

#### 3.2.4 Ventriculoperitoneal Shunt

In the analysis of three studies, neuroendoscopy was associated with a lower ventriculoperitoneal shunt rate compared with EVD (posterior RR 0.48, 95% CrI 0.25–0.91) (Figure S15). Heterogeneity was low (posterior median *I* ^2^ = 23.4%) (Figures S16 and S19). The posterior probability that neuroendoscopy reduces shunt requirement relative to EVD was 98.6% (Figures S17 and S18).

#### 3.2.5 Length of ICU Stay

The analysis of three studies showed that the neuroendoscopy group was associated with a shorter ICU stay compared with EVD (posterior MD –1.63 days, 95% CrI –5.49 to 2.31) (Figure S20). Between‐study variability was low (posterior median *I* ^2^ = 8.3%) (Figures S20 and S23). The posterior probability of benefit for neuroendoscopy was 79.7%, suggesting a moderate probability of reduced ICU length of stay, although uncertainty remains given that the credible interval included the null value (Figures S21 and S22).

#### 3.2.6 Sensitivity Analysis

Sensitivity analyses using a more informative prior (normal distribution with mean 0 and standard deviation 1) for the log risk ratio yielded results consistent with the primary model.

For mortality, the posterior risk ratio remained stable (RR 0.64, 95% CrI 0.32–1.28) compared with the primary estimate (RR 0.61), with minimal change in heterogeneity (posterior median *I* ^2^ 24.4% vs. 24.7%) (Figure S7) and posterior probability of benefit (89.9% vs. 90.9%).

Similarly, for rebleeding, effect estimates varied minimally under the alternative prior (RR 0.65, 95% CrI 0.29–1.44 vs. RR 0.61) (Figure S3), with comparable heterogeneity (posterior median *I* ^2^ 19.7% vs. 20.3%) (Figure 2 and S2) and identical posterior probability of benefit (87.4%).

#### 3.2.7 Subgroup Analyses

We ran subgroup analyses based on the study design to assure the consistency of our findings. All the results were consistent with the primary analysis (Figures S24–S26). We could not run subgroup analyses on ICU stay and ventriculoperitoneal shunt rate due to limited number of studies.

### 3.3 Meta‐Regression

There were not significant associations between treatment effect and mean age (*B*_1_ = 0.10; *p* = 0.16; Figure S27), preoperative Glasgow Coma Scale (GCS) score (*B*_1_ = 0.25; *p* = 0.25; Figure S28), or mean intracranial hematoma volume (*B*_1_ = –0.32; *p* = 0.08; Figure S29) for mortality rate. There was not association between these covariates with rebleeding and prognosis (Figures S27–S35.

### 3.4 Risk of Bias Assessment and Summary of Findings

**FIGURE 5:**
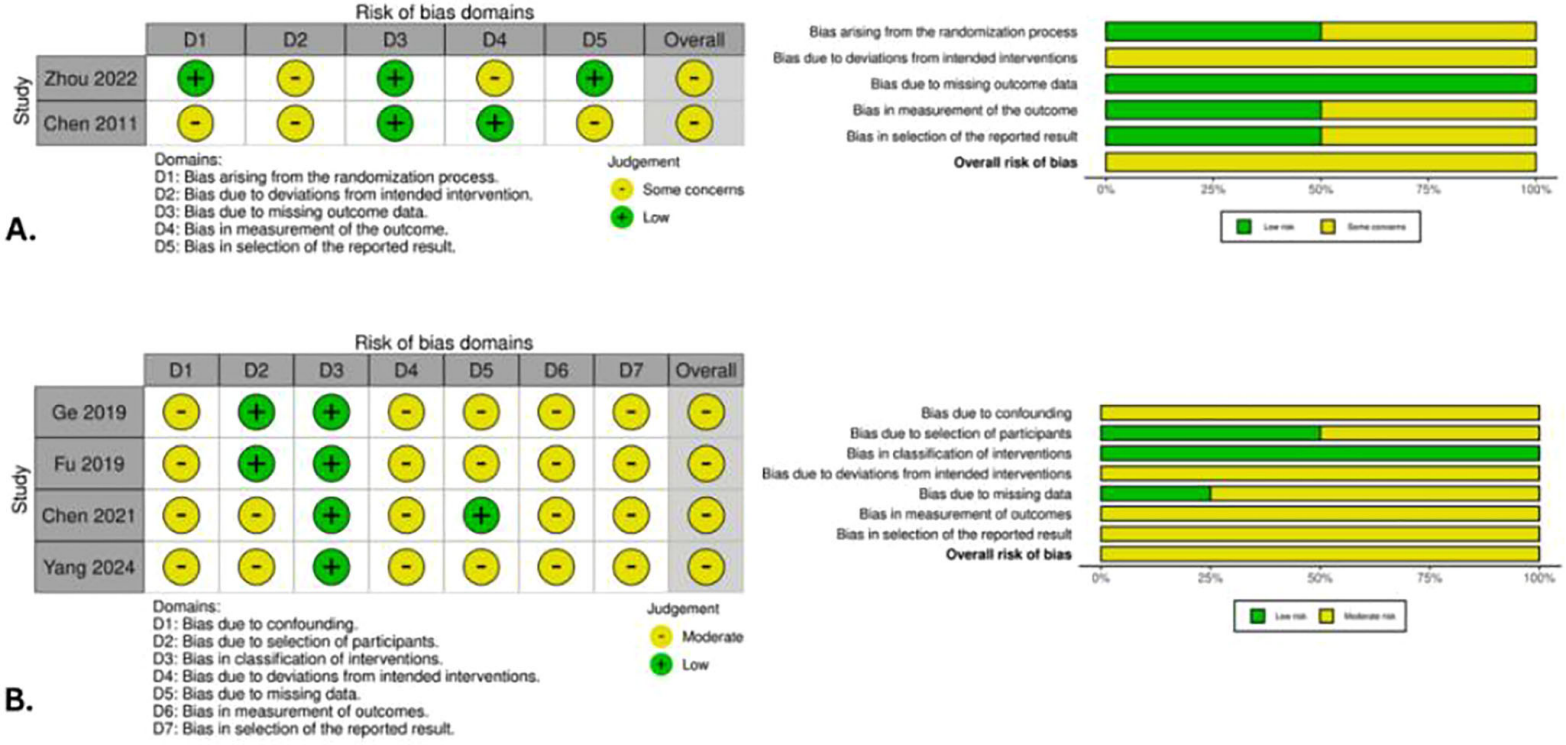
RoB 2.0 risk of bias scoring traffic light plot and weighted bar plot for randomized clinical trials included in the final analysis of this review (A). ROBINS‐I risk of bias scoring traffic light plot and weighted bar plot for non‐randomized clinical studies included in the final analysis of this review (B).

The quality assessment of included studies is presented in Figure 5. Overall, both randomized and nonrandomized trials demonstrated a moderate risk of bias, primarily due to confounding, deviations from intended interventions, missing data, and selective reporting. Although powerless, we present the results of publication bias in the Supplementary material (Figures S4 and S11).

Certainty of evidence was evaluated according to risk of bias, indirectness, imprecision, publication bias, and inconsistency. All outcomes were rated as very low certainty due to concerns present in all included studies (Table 2).

**TABLE 2:** The certainty of evidence for each outcome was evaluated considering risk of bias, inconsistency, indirectness, imprecision, and publication bias, and graded as high, moderate, low, or very low certainty.

Outcome	No. of studies	Study design	Risk of bias	Inconsistency	Indirectness	Imprecision	Other considerations	Neuroendoscopy	External ventricular drainage	Effect estimate	Certainty of evidence	Importance
Mortality	6	Non‐randomized studies	Serious	Not serious	Not serious	Serious	None	17/195 (8.7%)	31/204 (15.2%)	RR 0.61 (95% CI 0.30–1.27)	⨁◯◯◯ Very Low	Critical
ICU length of stay	3	Non‐randomized studies	Serious	Not serious	Not serious	Very serious	None	102 patients	102 patients	MD −1.63 days (95% CI −5.49 to 2.31)	⨁◯◯◯ Very Low	Critical
Prognosis	5	Non‐randomized studies	Serious	Serious	Not serious	Serious	None	96/175 (54.9%)	99/184 (53.8%)	RR 1.04 (95% CI 0.67–1.60)	⨁◯◯◯ Very Low	Critical
Rebleeding	5	Non‐randomized studies	Serious	Not serious	Not serious	Serious	None	14/175 (8.0%)	25/184 (13.6%)	RR 0.61 (95% CI 0.26–1.46)	⨁◯◯◯ Very Low	Critical
Ventriculoperitoneal shunt rate	3	Non‐randomized studies	Serious	Not serious	Not serious	Not serious	None	17/51 (33.3%)	36/51 (70.6%)	RR 0.48 (95% CI 0.25–0.91)	⨁◯◯◯ Very Low	Critical

## 4 Discussion

In this systematic review and Bayesian meta‐analysis, we synthesized contemporary evidence comparing neuroendoscopy with external ventricular drainage (EVD) for the management of thalamic hemorrhagic stroke. Overall, neuroendoscopy was associated with directional reductions in mortality, rebleeding risk, favorable and ICU length of stay. Importantly, neuroendoscopy was consistently associated with a significantly lower ventriculoperitoneal shunt requirement and favorable prognosis, representing the most robust findings of this meta‐analysis.

While the credible intervals for mortality and rebleeding included the null value, posterior probability estimates favored neuroendoscopy for these outcomes, the reduction in shunt dependency and favorable prognosis were statistically credible, reinforcing the clinical advantage of neuroendoscopy. Regarding rebleeding, although neuroendoscopy has been linked to improved outcomes overall, our analysis did not demonstrate a statistically significant difference between interventions. Nevertheless, despite the observed high probability of benefit, the low certainty of evidence and consistent comparable results in subgroup analyses warrants confirmation in adequately powered randomized trials.

The directional reduction in mortality observed here aligns with prior literature supporting minimally invasive approaches in deep intracerebral hemorrhage (Mezzacappa et al. 2023; Pramatya et al. 2025; Scaggiante et al. 2018; Sun et al. 2024). The recent meta‐analysis by Pramatya et al. (2025) demonstrated a significant reduction in mortality with minimally invasive surgery (MIS) compared with conventional procedures. Subgroup analyses in that study showed that neuroendoscopy was associated with lower mortality compared specifically with EVD, closely mirroring our findings. Similarly, the updated *Stroke* meta‐analysis of randomized controlled trials evaluating MIS in supratentorial ICH reported significant reductions in death and dependence, particularly within the neuroendoscopy subgroup (Scaggiante et al. 2018).

Neuroendoscopy is thought to lower mortality in ICH primarily by reducing hematoma volume, thereby alleviating mass effect and decreasing secondary brain injury, including perihematomal edema and inflammation (Pradilla et al. 2024; Scaggiante et al. 2018). neuroendoscopy enables rapid hematoma evacuation and hemostasis under direct visualization, thereby improving neurological outcomes without increasing complications (Fu et al. 2019).

The reduction in ventriculoperitoneal shunt placement observed in our analysis represents one of the most consistent and clinically meaningful finding. In thalamic hemorrhage, intraventricular extension is common and frequently leads to obstructive hydrocephalus requiring CSF diversion due to ventricular blood burden and impaired CSF circulation (Menon et al. 2019; Mezzacappa et al. 2023). Consistent with the findings of Mezzacappa et al., the need for permanent CSF diversion was less likely in patients treated with neuroendoscopy. This reduction is clinically relevant, as ventriculoperitoneal shunt placement carries substantial morbidity, including infection, shunt obstruction, overdrainage leading to subdural hematoma, and the need for revision procedures (Lu et al. 2019). The reduction in shunt dependency may partly explain the observed trends in ICU duration and survival, as persistent hydrocephalus is a well‐recognized driver of neurological deterioration and prolonged critical care.

The observed trend toward shorter ICU stays with neuroendoscopy, although not statistically significant, may represent a clinically meaningful signal. While the credible interval included the null value, the posterior probability of benefit approached 80%, suggesting a moderate likelihood of reduced ICU utilization. Prolonged ICU stay has been associated with increased in‐hospital mortality, higher complication rates, and greater healthcare resource consumption. Furthermore, Mezzacappa et al. (2023) reported shorter overall hospitalization in patients undergoing neuroendoscopy compared with EVD, supporting the hypothesis that definitive clot evacuation may facilitate faster physiological stabilization and recovery.

Although meta‐regression could not identify any association between outcomes and some covariates likely due to limited number of studies, clinical heterogeneity was evident across included studies in terms of patient characteristics, interventions, and outcome definitions. For example, mean hematoma volumes ranged from 10 to 35 mL, baseline GCS scores from 5.8 to 9.8, and follow‐up durations from 3 to 12 months. Neuroendoscopy techniques varied from conventional to smartphone‐assisted approaches, while EVD protocols were not standardized. Importantly, study designs differed: two randomized controlled trials contrasted with four retrospective or nonrandomized cohorts. This methodological heterogeneity, combined with clinical variability, may partly explain differences in effect estimates and underscores the need for adequately powered randomized trials to confirm the observed posterior probabilities of benefit.

This study has several important limitations. First, the pooled estimates are derived from a relatively small number of studies, resulting in limited statistical power and greater uncertainty in effect estimates. The small number of included studies also precluded formal assessment of publication bias, thereby limiting evaluation of potential small‐study effects. Additionally, many of the included studies were retrospective or prospective but nonrandomized, introducing potential selection bias, residual confounding, and variability in baseline characteristics that could influence outcomes. Therefore, the findings should be interpreted cautiously until confirmed by adequately powered randomized controlled trials.

## 5 Conclusion

In this Bayesian meta‐analysis of randomized and nonrandomized studies of patients with thalamic hemorrhage, neuroendoscopy was associated with lower ventriculoperitoneal shunt rate and favorable prognosis compared with external ventricular drainage. However, these findings should be considered as hypothesis‐generating for future studies, rather than practice‐changing as due to smaller number of included studies, low certainty, and imprecision. These results underscore the need for adequately powered randomized controlled trials to confirm the survival and functional benefits suggested by this analysis.

## Author Contributions

**Pedro Tchicama Sikembi**: methodology, formal analysis, supervision, writing – review and editing. **Pablo Andrés Vega‐Medina**: data curation. **Albe Dias Batista**: visualization. **Davi Ricardo Soares Gama de Amorim**: data curation. **Angel F. Godina‐Sanchez**: validation. **Laura Alexandra González‐Chang**: investigation, writing – original draft.

## Funding

The authors have nothing to report.

## Ethics Statement

This study did not involve direct human participants. Ethical approval was not required as the analysis was based on previously published data.

## Conflicts of Interest

The authors declare no conflicts of interest.

## Supporting information




**Supplementary Materials**: brb371526‐sup‐0001‐SuppMat.docx

## Data Availability

All data supporting the findings of this study are available within the paper and its Supplementary Information.
