# Supplementary material for: Neuroendoscopy Versus External Ventricular Drainage for Thalamic Hemorrhagic Stroke: A Systematic Review and Bayesian Meta‐Analysis
Source: Brain Behav. 2026 Jun 21;16(6):e71526. doi: 10.1002/brb3.71526 (PMC13283911; doi:10.1002/brb3.71526)

**Supplementary Material 1**

**Table S1.** Complete Search Strategy for PubMed, Embase and Cochrane Databases

| **Database** | **Search strategy** |
| --- | --- |
| PubMed (n=182) | ("Intracranial Hemorrhages"[Mesh] OR ``Thalamic Hemorrhage´´ OR ``Thalamic Hematoma´´ OR ``Thalamic Stroke´´ OR ``Thalamic Bleed´´) AND (``Minimally Invasive Surgery´´ OR "Minimally Invasive Surgical Procedures"[Mesh] OR "Neuroendoscopy"[Mesh] OR ``Neuroendoscopic Surgery´´) AND (``Ventricular Drainage´´ OR EVD OR "Ventriculostomy"[Mesh] OR ``External Ventricular Drainage´´) |
| Web of Sciences (n=10) | ("Intracranial Hemorrhages" OR “Thalamic Hemorrhage” OR “Thalamic Hematoma” OR “Thalamic Stroke” OR “Thalamic Bleed”) AND (“Minimally Invasive Surgery” OR “Minimally Invasive Surgical Procedures” OR Neuroendoscopy OR “Neuroendoscopic Surgery”) AND (“Ventricular Drainage” OR EVD OR Ventriculostomy OR “External Ventricular Drainage”) |
| EMBASE (n=155) | ("Intracranial Hemorrhages" OR “Thalamic Hemorrhage” OR “Thalamic Hematoma” OR “Thalamic Stroke” OR “Thalamic Bleed”) AND (“Minimally Invasive Surgery” OR “Minimally Invasive Surgical Procedures” OR Neuroendoscopy OR “Neuroendoscopic Surgery”) AND (“Ventricular Drainage” OR EVD OR Ventriculostomy OR “External Ventricular Drainage”) |

**Rebleeding**

**Figure S1.** Full posterior distribution of the estimated Log risk ratio


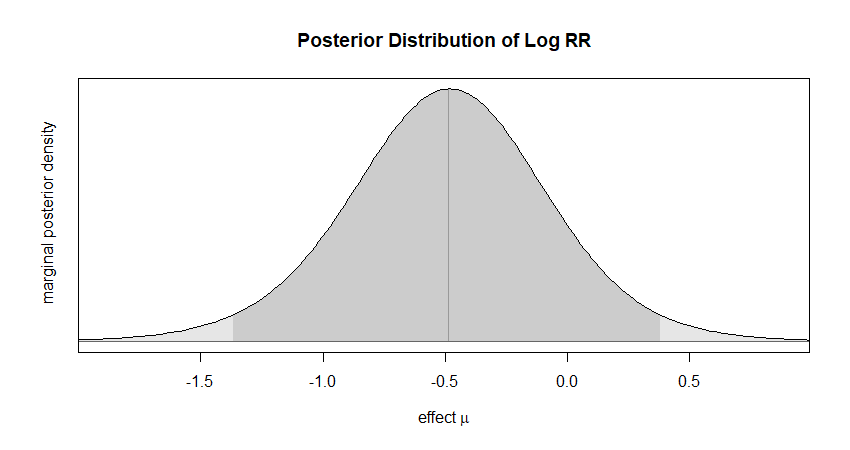


**Figure S2:** Joint posterior distribution of the estimated Log risk ratio and heterogeneity


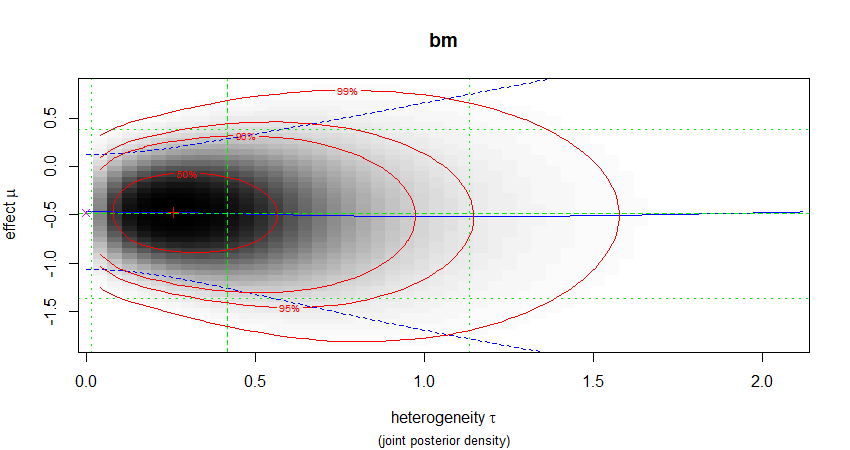


**Figure S3.** Forest plot comparing neuroendoscopy versus external ventricular drainage by applying a weakly informative prior

**
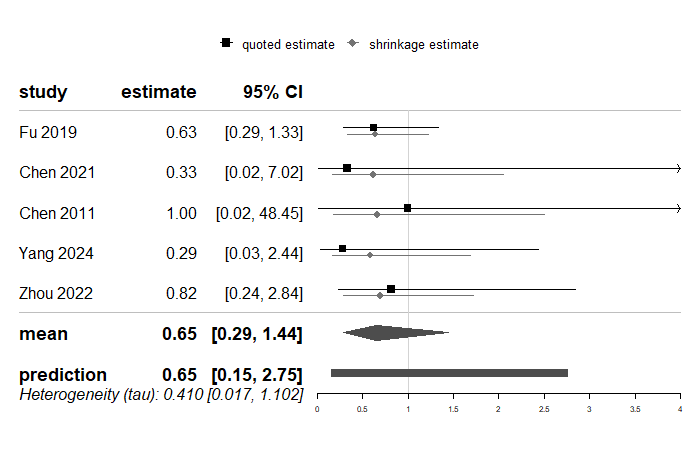
**

**Figure S4.** Funnel plot for visual assessment of publication bias

**
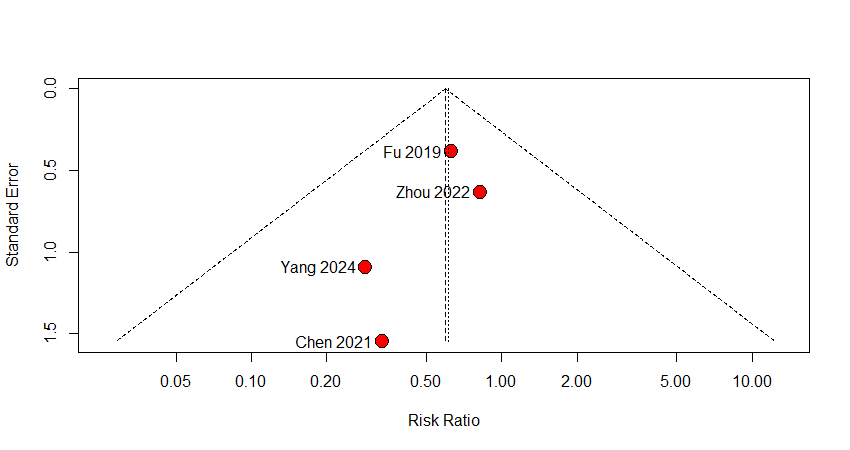
**

**Mortality**

**Figure S5**. Bayesian forest plot comparing neuroendoscopy versus external ventricular drainage for the primary analysis
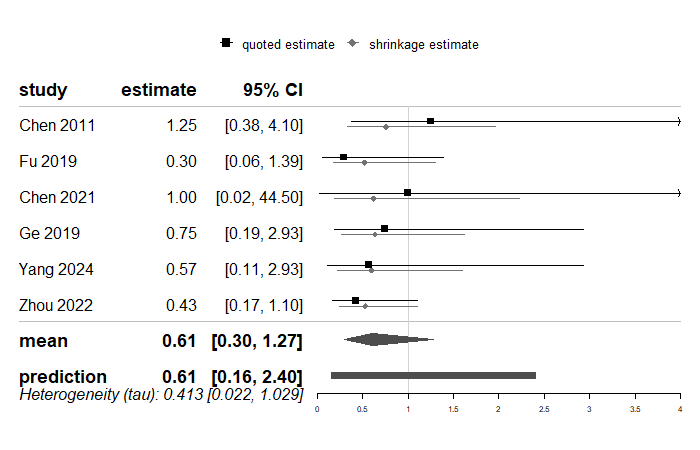


**Figure S6.** Frequentist forest plot comparing neuroendoscopy versus external ventricular drainage


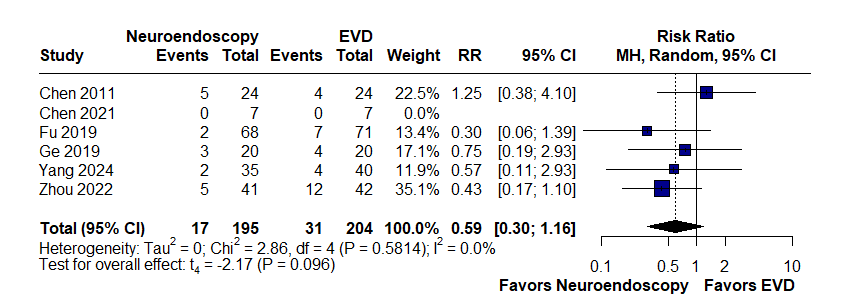


**Figure S7.** Bayesian forest plot comparing neuroendoscopy versus external ventricular

drainage by applying a weakly informative prior


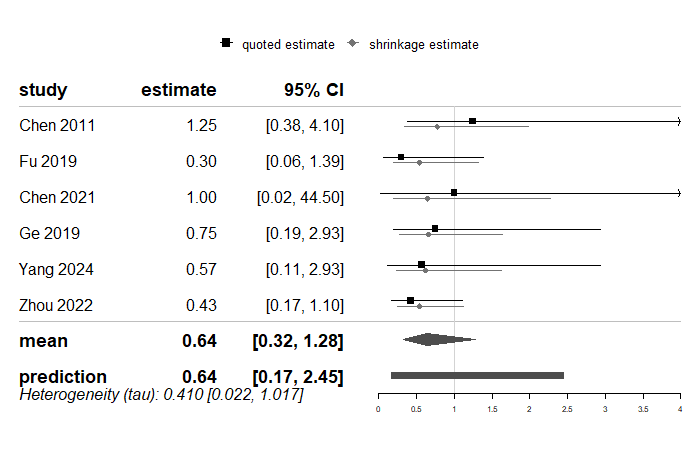


**Figure S8.** Cumulative posterior distribution of the estimated Log risk ratio

**
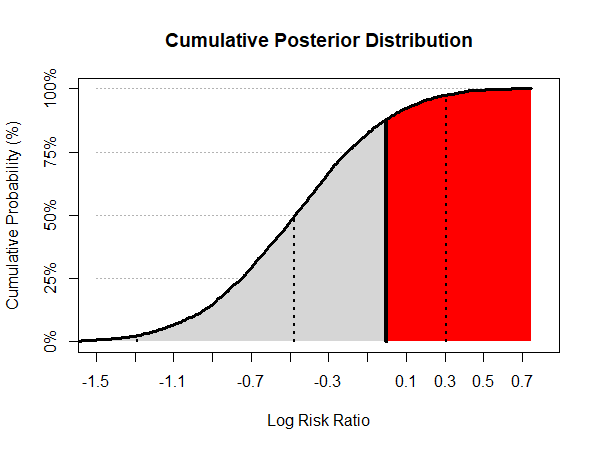
**

**Figure S9.** Full posterior distribution of the estimated Log risk ratio


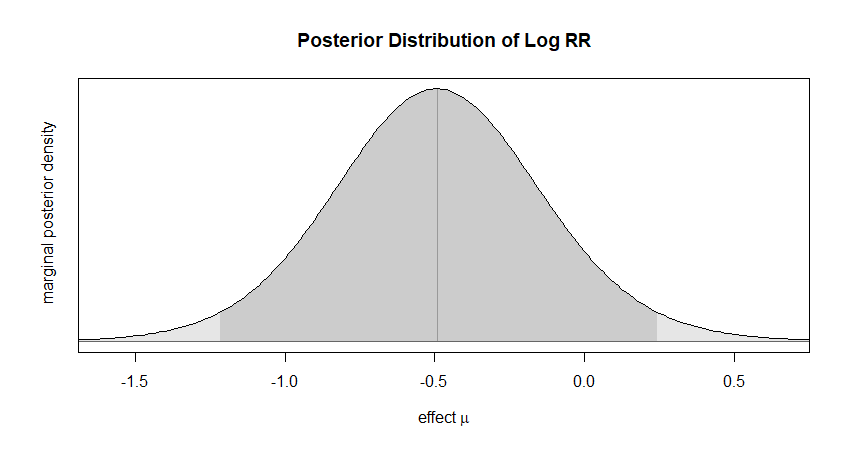


**Figure S10.** Joint posterior distribution of the estimated Log risk ratio and heterogeneity


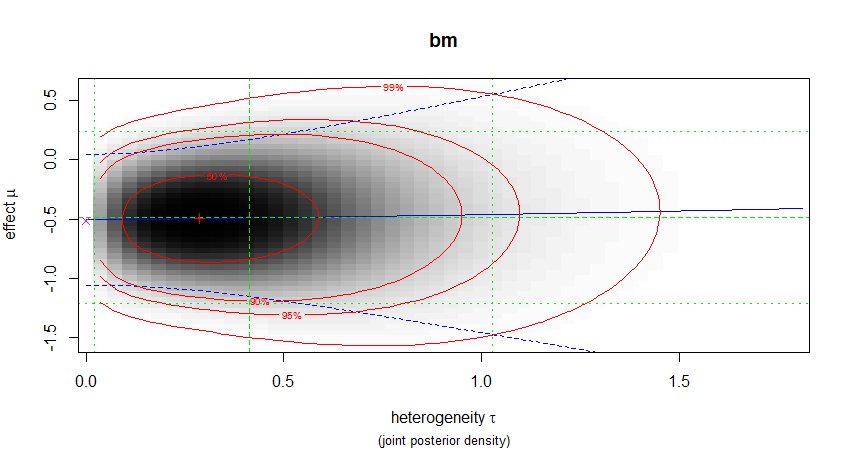


**Figure S11.** Funnel plot for visual assessment of publication bias


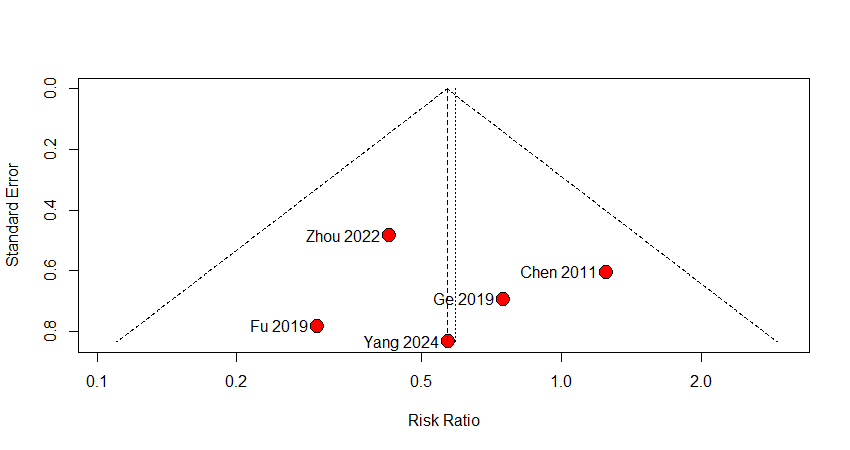


**Prognosis**

**Figure S12.** Cumulative posterior distribution of the estimated Log risk ratio


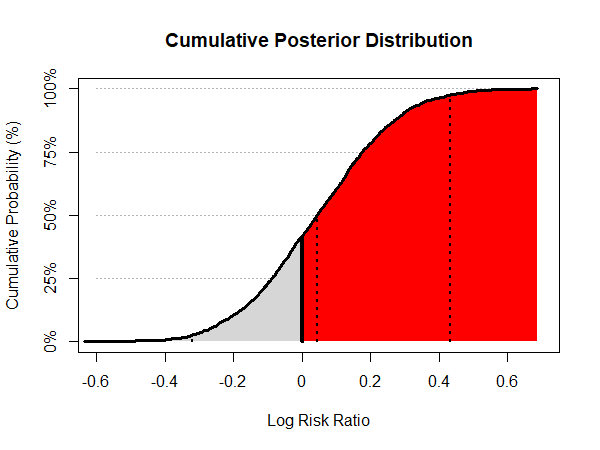


**Figure S13.** Full posterior distribution of the estimated Log risk ratio


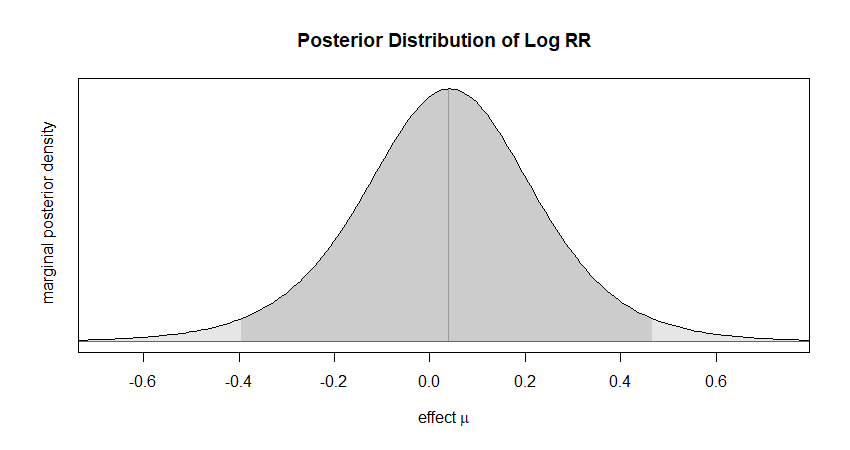


**Figure S14.** Joint posterior distribution of the estimated Log risk ratio and heterogeneity


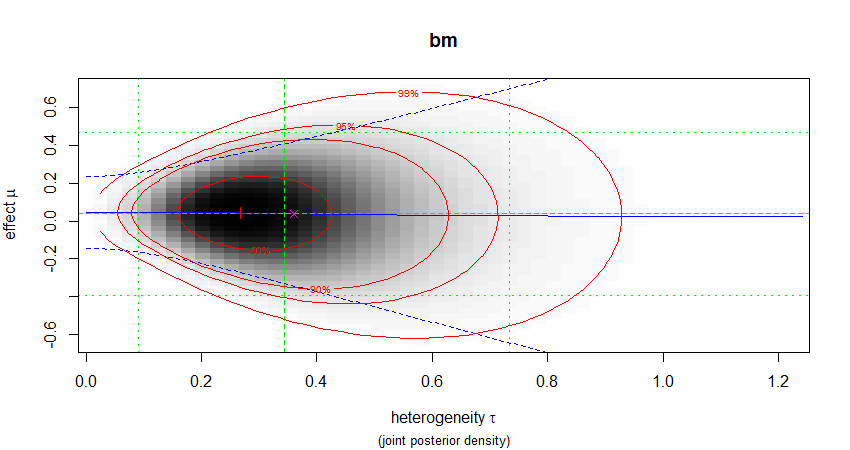


**Ventriculoperitoneal shunt**

**Figure S15.** Prognostic outcomes comparing neuroendoscopy and external ventricular drainage (EVD)


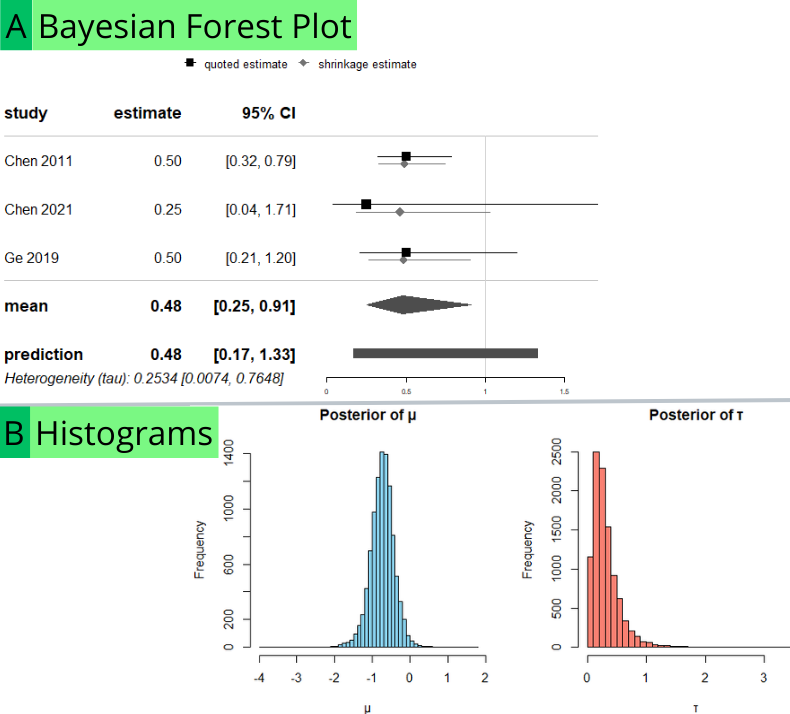


(A) Bayesian forest plot showing study-level and pooled risk ratios (RR) with 95% credible intervals. (B) Posterior distributions for the overall treatment effect (μ) and between-study heterogeneity (τ).

**Figure S16**. Bayesian forest plot comparing neuroendoscopy versus external ventricular drainage for the primary analysis


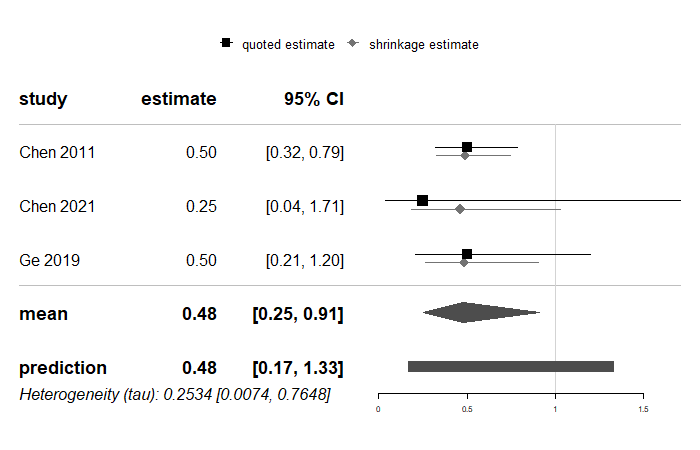


**Figure S17.** Cumulative posterior distribution of the estimated Log risk ratio


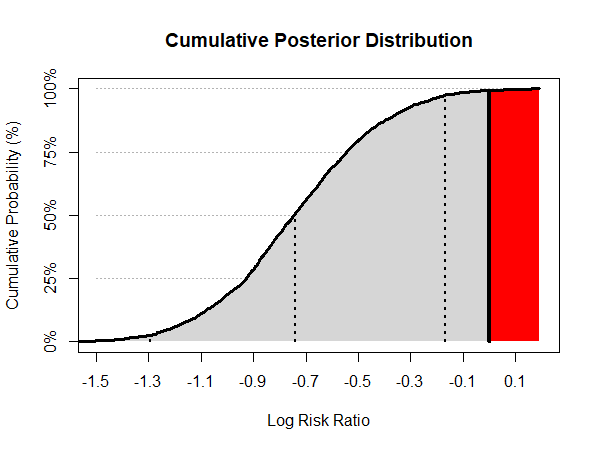


**Figure S18.** Full posterior distribution of the estimated Log risk ratio


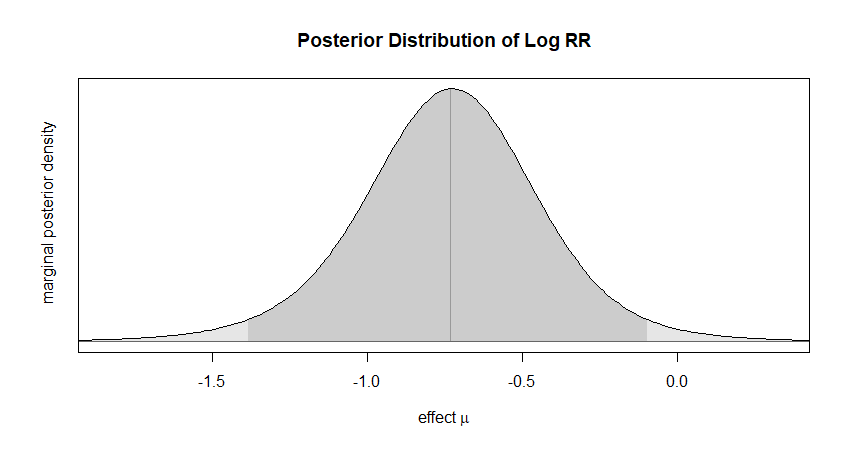


**Figure S19.** Joint posterior distribution of the estimated Log risk ratio and heterogeneity


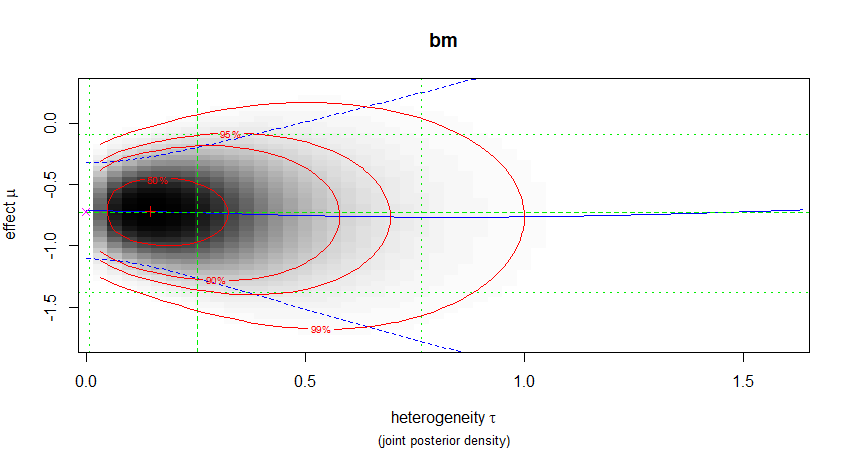


**ICU stay**

**Figure S20**. Bayesian forest plot comparing neuroendoscopy versus external ventricular drainage for the primary analysis


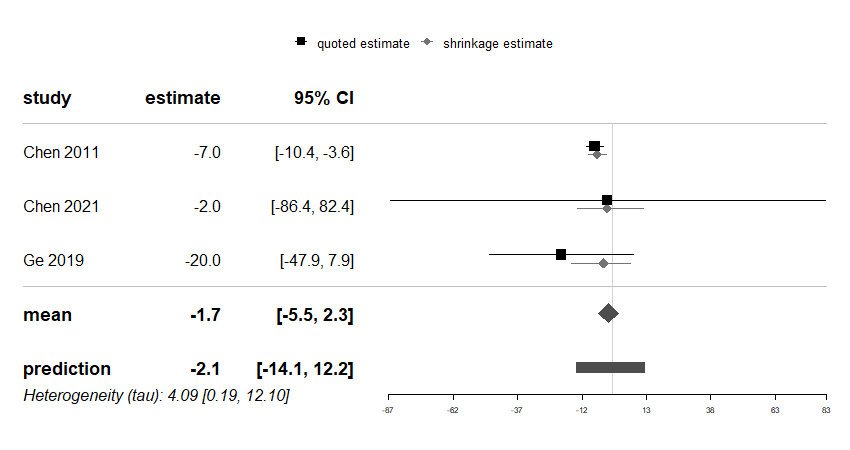


**Figure S21.** Cumulative posterior distribution of the estimated Log risk ratio


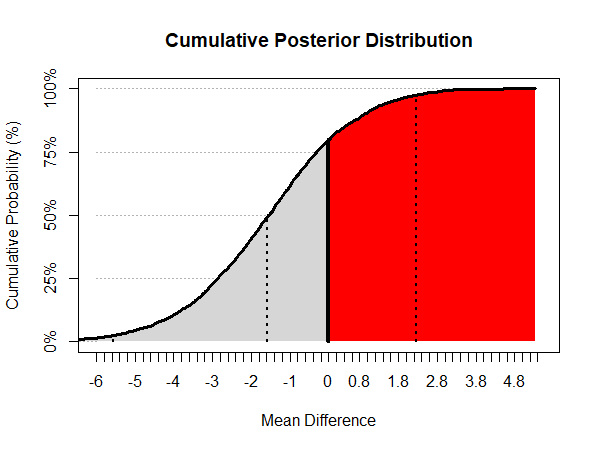


**Figure S22.** Full posterior distribution of the estimated Log risk ratio


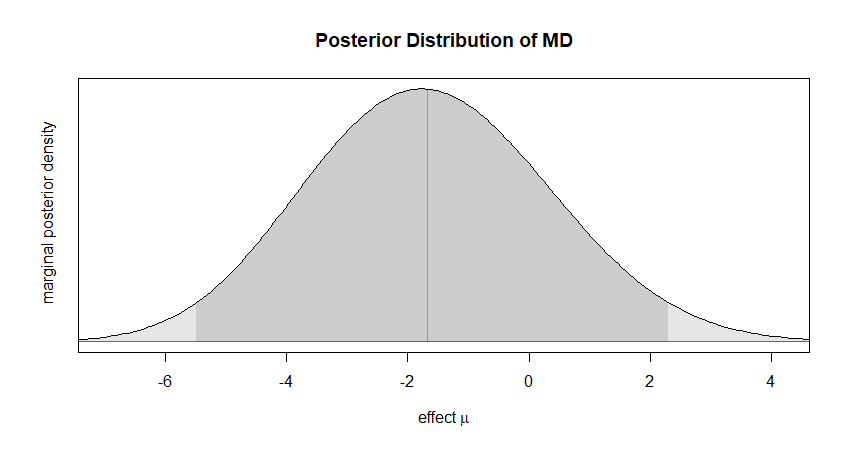


**Figure S23.** Joint posterior distribution of the estimated Log risk ratio and heterogeneity


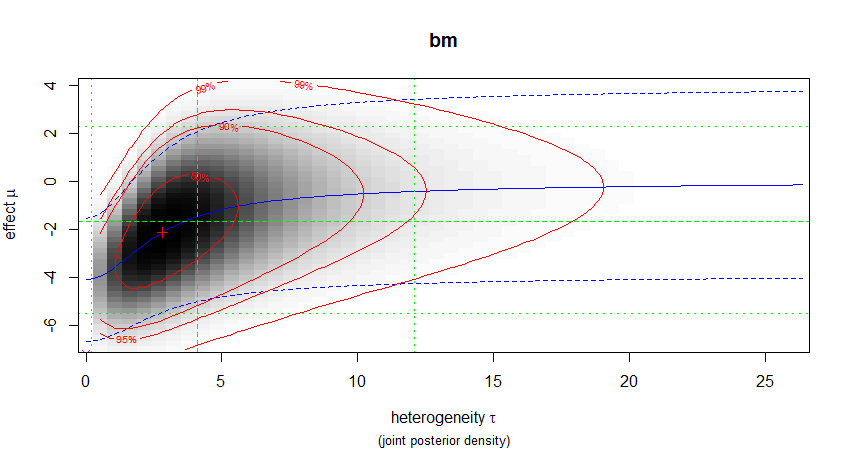


Subgroup-Analyses Based on Study Design

Figure S24: Rebleeding


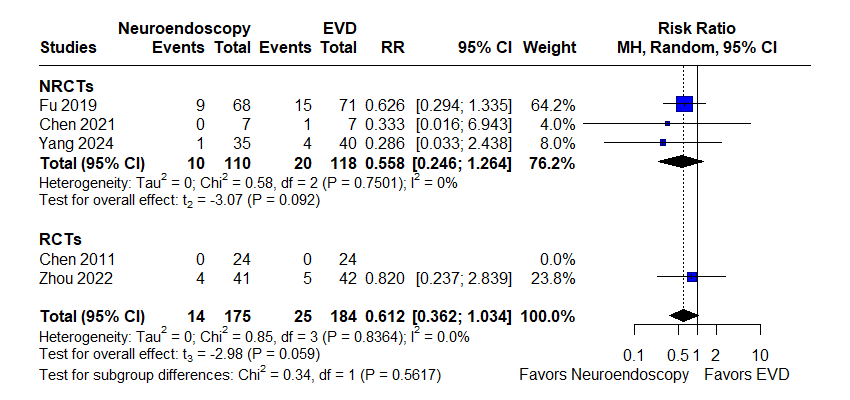


Figure S25: Mortality


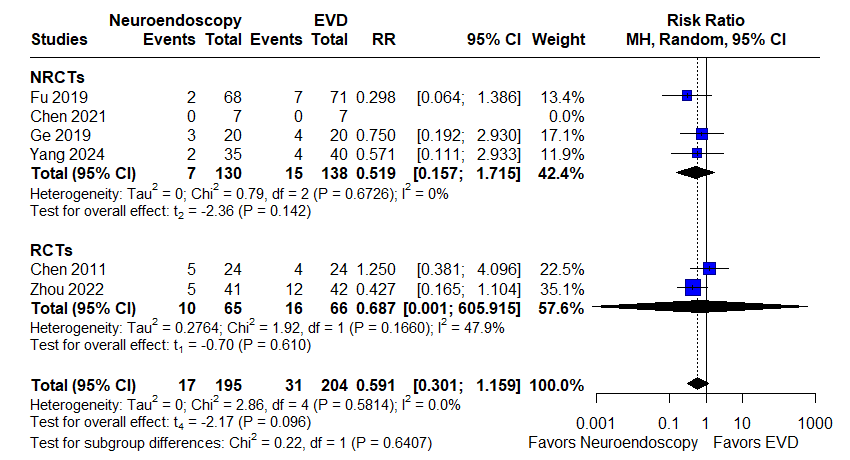


Figure S26: Prognosis


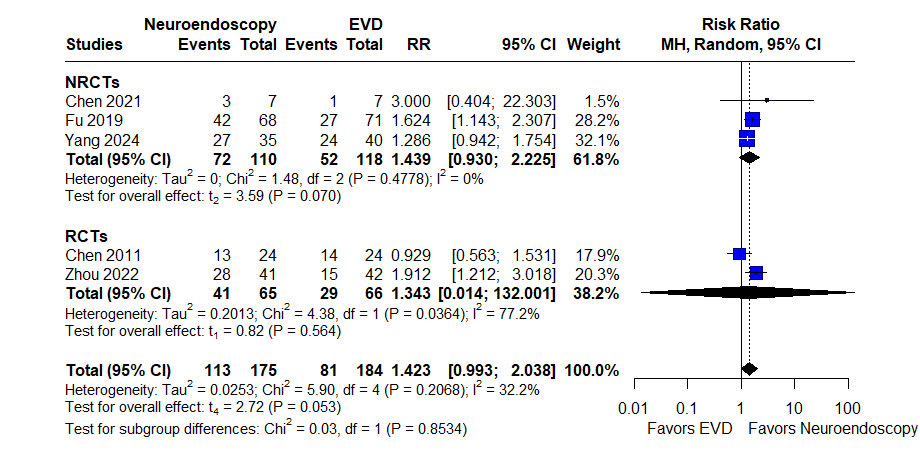


**Additional analysis: Meta-regression**

**Mortality**

Figure S27: Bubble plot illustrating no statistically significant influence of mean age on mortality


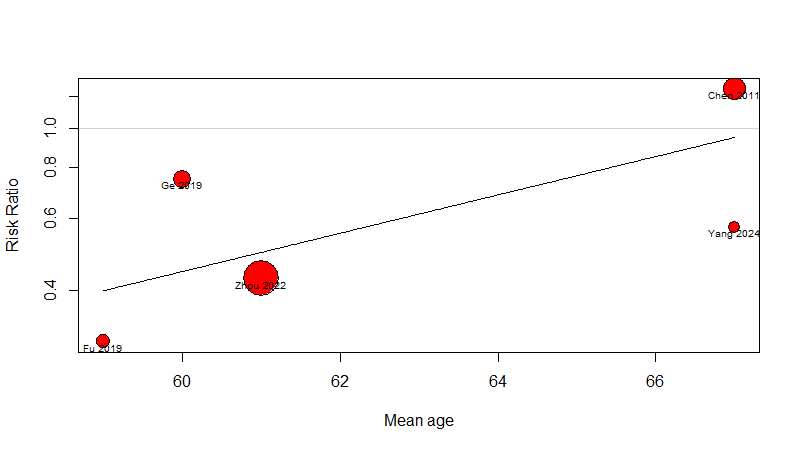


Figure S28: Bubble plot illustrating no statistically significant influence of Glasgow Coma Scale scores on mortality


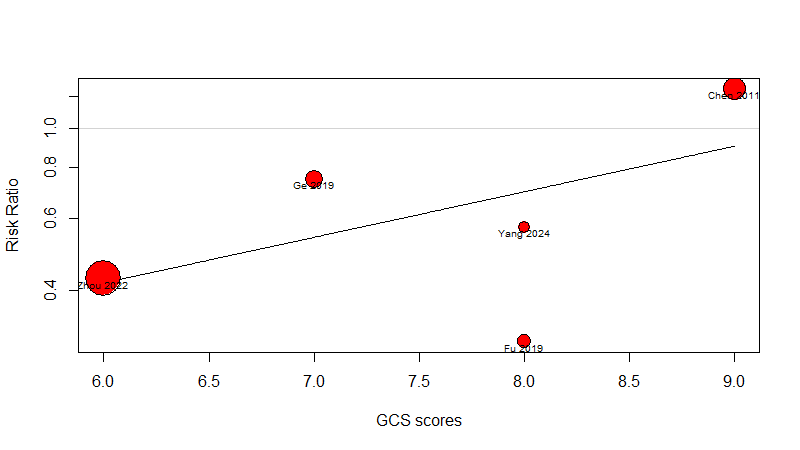


Figure S29: Bubble plot illustrating no statistically significant influence of mean follow-up duration on mortality


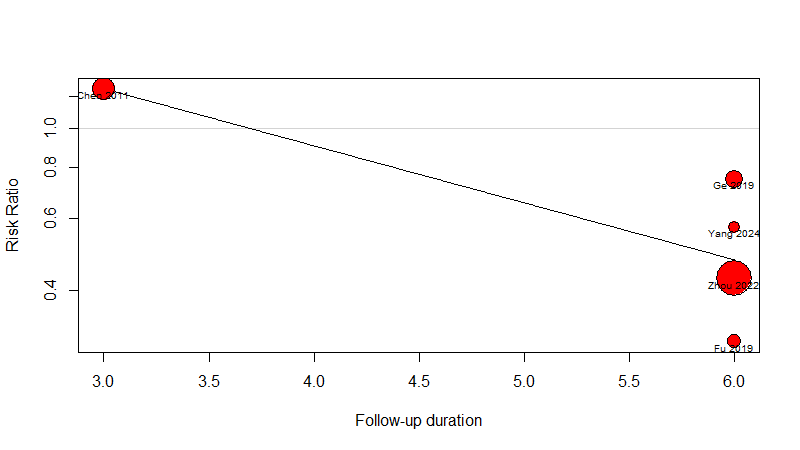


**Rebleeding**

Figure S30: Bubble plot illustrating no statistically significant influence of Glasgow Coma Scale scores on rebleeding


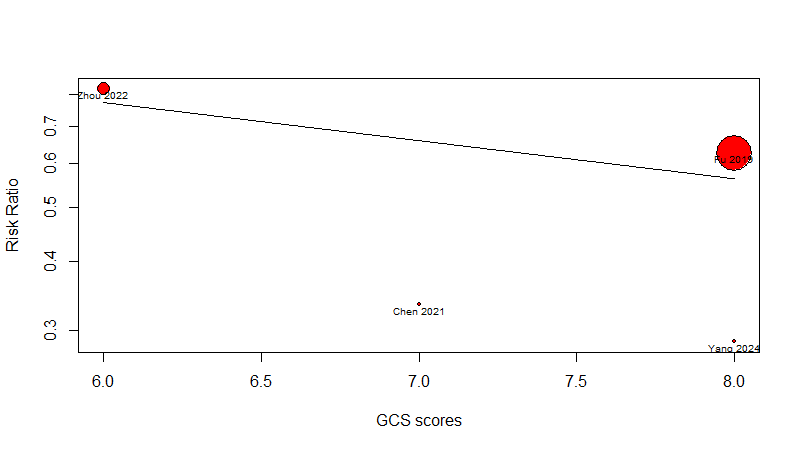


Figure S31: Bubble plot illustrating no statistically significant influence of mean age on rebleeding


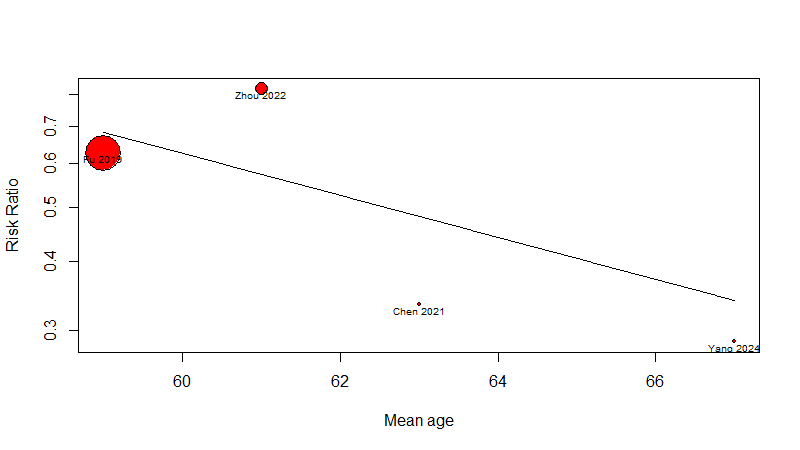


Figure S32: Bubble plot illustrating no statistically significant influence of mean follow-up duration on rebleeding


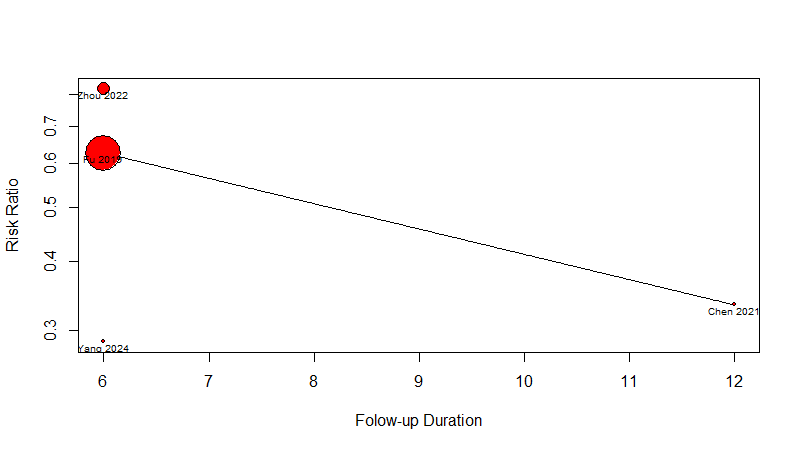


**Prognosis**

Figure S33: Bubble plot illustrating no statistically significant influence of Glasgow Coma Scale scores on prognosis


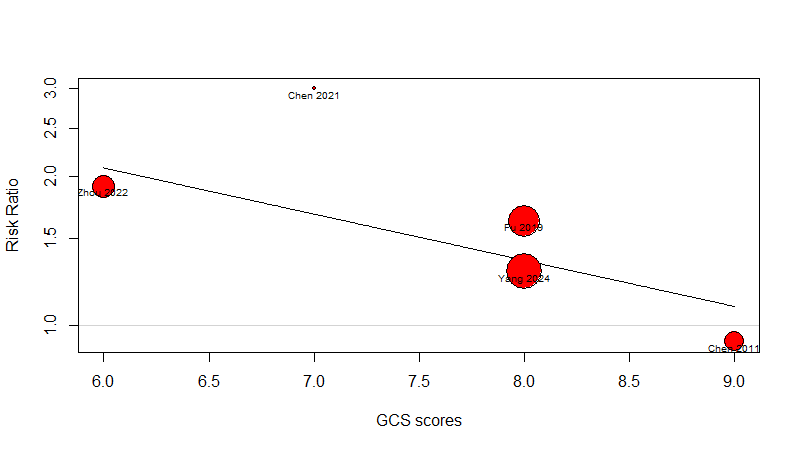


Figure S34: Bubble plot illustrating no statistically significant influence of mean age on prognosis


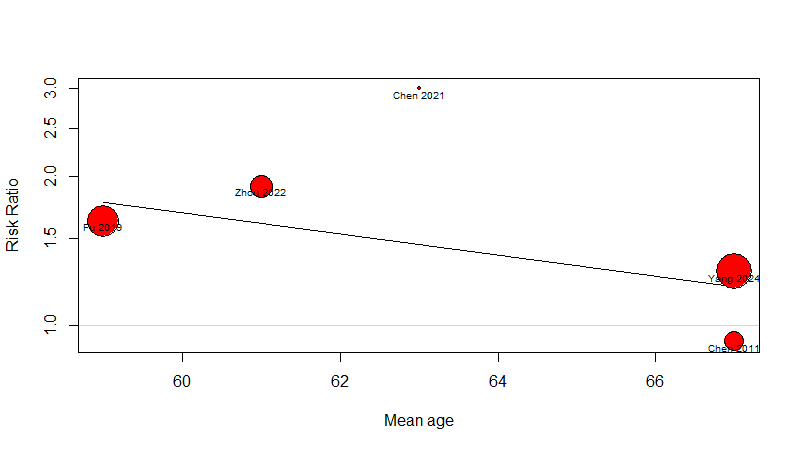


Figure S35: Bubble plot illustrating no statistically significant influence of mean follow-up duration on prognosis


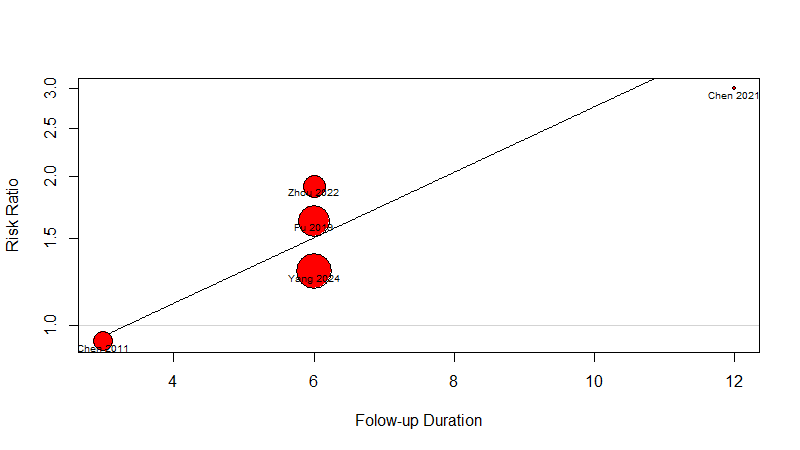

Supplement: Supplementary file 1 — Supplementary Materials: brb371526‐sup‐0001‐SuppMat.docx [file BRB3-16-e71526-s001.docx]
